# Association between triglyceride-glucose index and depression in patients with type 2 diabetes: A cross-sectional study from NHANES

**DOI:** 10.1097/MD.0000000000039258

**Published:** 2024-08-09

**Authors:** Jiaju Ren, Cheng Lv, Jia Wang

**Affiliations:** a School of Traditional Chinese Medicine, Beijing University of Chinese Medicine, Beijing, China; b School of Management, Beijing University of Chinese Medicine, Beijing, China; c Dongzhimen Hospital Beijing University of Chinese Medicine, Beijing, China; d General Medicine Department, Emergency General Hospital, Beijing, China.

**Keywords:** depression, triglyceride-glucose index, type 2 diabetes

## Abstract

This cross-sectional study aimed to examine the association between the triglyceride-glucose (TyG) index and the prevalence of depression in individuals with type 2 diabetes. A nationally representative sample of 3225 individuals with type 2 diabetes was enrolled in this study. Multivariable logistic regression models were used to assess the association between the TyG index and depression, adjusting for potential confounding factors. After adjusting for age, gender, BMI, smoking, alcohol consumption, congestive heart failure, and coronary heart disease, a significant positive association was found between the TyG index and the prevalence of depression in individuals with type 2 diabetes (OR = 1.54, 95% CI: 1.21–1.95). Subgroup analyses showed consistent associations across various demographic and clinical subgroups. This study provides evidence of a significant independent positive association between the TyG index and the prevalence of depression in individuals with type 2 diabetes.

## 1. Introduction

Depression, a significant global health problem, is characterized by persistent and pronounced low mood symptoms, imposing a substantial burden on individuals and society. It leads to a reduced quality of life, increased healthcare utilization, and even elevated mortality. A research study conducted on the U.S. population indicates that about 19.7% adults reported symptoms of depression at least once a month.^[1]^ The World Health Organization projects that depression will become a significant burden of disease by 2030.^[2]^ Various studies have established a connection between depression and several disease states, including cardiovascular disease, neurological disorders, and metabolic disorders.^[3–5]^ Additionally, as one of the most common disorders globally, depression among people with diabetes has different risks of onset and disease characteristics. For example, in the type 2 diabetic population, the prevalence of depression is nearly twice as high as in the general population, with the highest prevalence rate reaching approximately 30%.^[6]^ Furthermore, depression in this population is more severe compared to the general population, contributing to challenges in glycemic control, an increased risk of diabetic complications, and higher mortality.^[7–9]^ Consequently, further research is necessary to evaluate and comprehend the risk factors associated with depression in the type 2 diabetic population.

The triglyceride-glucose (TyG) index is a quantitative indicator that measures the relationship between fasting plasma triglyceride (TG) and glucose levels. In recent years, it has gained significant attention in research and clinical practice due to its potential as a novel tool for assessing metabolic disorders, particularly those related to type 2 diabetes.^[10–12]^ Several studies conducted in China have demonstrated an association between TyG index and an increased prevalence of type 2 diabetes among adults, as well as a rise in complications associated with type 2 diabetes in hospitalized patients.^[12,13]^ Furthermore, the TyG index has been found to be a predictor of all-cause mortality and cardiovascular mortality in individuals with diabetes.^[14–16]^ It has also been associated with the prevalence of coronary artery stenosis, atherosclerosis, retinopathy, and nonalcoholic fatty liver disease in this population.^[17–20]^ However, the current understanding of the association between TyG index and the prevalence of depression in individuals with type 2 diabetes is unclear. Further research is needed to explore this relationship. The primary objective of this study is to explore the independent association between baseline TyG index and the prevalence of depression in individuals with type 2 diabetes. The research aims to supplement existing literature by utilizing the comprehensive 2005 to 2020 National Health and Nutrition Examination Survey (NHANES) dataset for investigation purposes.

## 2. Methods

### 2.1. Study design and population

This study utilized data from the NHANES, a nationally representative survey conducted periodically by the Centers for Disease Control and Prevention (CDC). NHANES provides comprehensive data on the health and nutritional status of the population, making it a valuable resource for determining disease prevalence and trends. The survey’s robust methodology and large sample size make it an essential tool for public health research.

**Figure 1.**
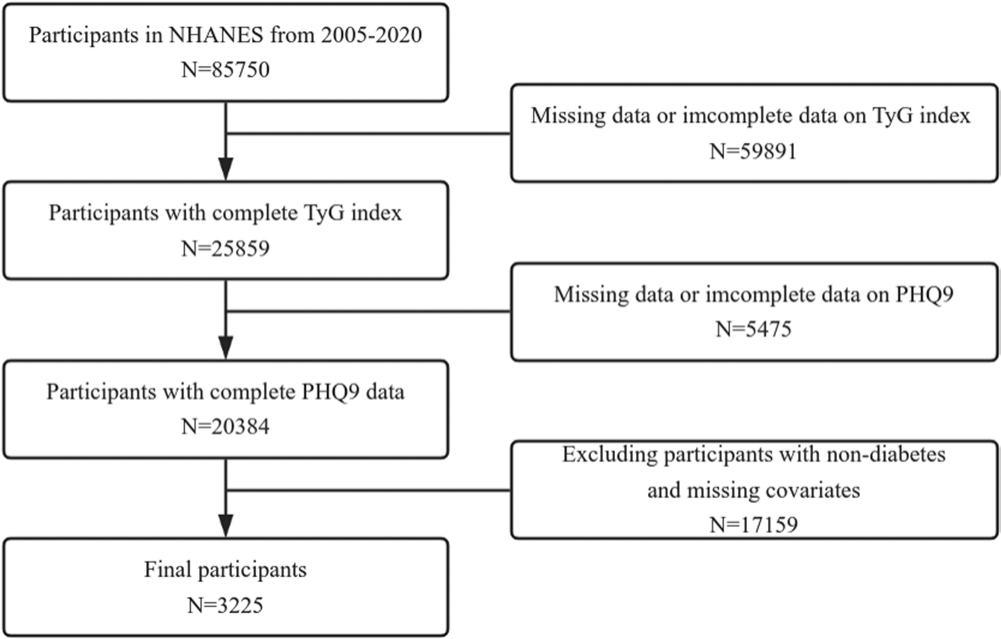
Flow chart of participant selection.

For our study, we included data from all 8 cycles of NHANES, spanning the years 2005 to 2020. Our study population consisted of individuals aged 18 years and older. To ensure data quality, type 2 diabetic participants with missing values for TyG, Patient Health Questionnaire-9 (PHQ-9), and other study variables were excluded from the analyses. This process resulted in a final sample size of 3225 participants (Fig. 1).

### 2.2. Primary research variables

Plasma glucose and serum triglyceride levels were measured using an automated analyzer. The TyG index, which quantifies the relationship between fasting triglycerides (mg/dL) and fasting glucose (mg/dL), was calculated using the formula:

TyG = Ln[fasting triglycerides (mg/dL) × fasting glucose (mg/dL)/2]. For the purpose of analyses, the TyG index was categorized into 4 groups based on quartiles: Q1 < 8.61, Q2 (8.61,9.03), Q3 (9.03,9.47), and Q4 (>9.47). Participants’ depressive status was assessed using the PHQ-9. A PHQ-9 score of ≥10 indicated the presence of depression, and individuals meeting this criterion were categorized as depressed. Conversely, those below the threshold were considered nondepressed. Type 2 diabetes was defined based on the following criteria: fasting blood glucose ≥ 126mg/dL, hemoglobin A1c ≥ 5.29%, use of hypoglycemic therapy, or self-reported history of diabetes.^[21,22]^

### 2.3. Covariate

Based on the literature, we considered several potential confounders that may influence the association between TyG index and the risk of depression. These confounders include age, sex, race, marital status, body mass index (BMI), blood glucose level, triglyceride level, high-density lipoprotein (HDL) level, low-density lipoprotein (LDL) level, smoking habits, alcohol consumption, chronic heart failure (CHF), and coronary artery disease (CAD). BMI was categorized as follows: normal (<25.0), overweight (25.0–29.9), and obese (>30.0). Smoking status was classified as nonsmokers, former smokers, and current smokers. Alcohol consumption was categorized as none, moderate, and heavy, based on the average weekly alcohol intake (0, 1–8, and ≥8 drinks) in the past 12 months. The diagnosis of cardiovascular disease was based on a questionnaire or relevant medical history information. All these potential confounders were included in the analyses to account for their potential influence on the association between TyG index and the risk of depression.

### 2.4. Statistical analyses

The continuous data are expressed as weighted means (standard errors), whereas the categorical data are presented as weighted percentages. To eliminate the influence of confounding factors, multiple adjusted models were used to calculate the incidence rate of depression in the type 2 diabetic population based on the TyG index. The first model included only the TyG index. The second model adjusted for age, gender, race, and marital status. The third model adjusted for variables included in the second model, as well as BMI, smoking, alcohol consumption, CHF, and CAD. The results of the 3 models were presented as odds ratios (OR) and 95% confidence intervals (CI). To describe the dose–response relationship between the TyG index and the incidence of depression in the type 2 diabetic population, restricted cubic splines (RCS) were utilized. Subgroup analyses were conducted to explore the possible impact of demographic and clinical factors on the association between TyG index and depression. Subgroup factors included age, gender, marital status, BMI, smoking, alcohol consumption, history of CHF, and history of CAD. Interaction between subgroups was tested using the likelihood ratio test.

The analyses was conducted using the recommended weights of the database, and R (version 4.2.1) was used for all data processing and analyses. A significance level of *P* < .05 was considered statistically significant.

## 3. Results

### 3.1. Baseline characteristics of participants

The study population consisted of 3225 participants with type 2 diabetes, of which 1661 were males and 1564 were females. Among the participants, 2861 did not have depression, while 364 had depression. In Table 1, the baseline characteristics of the type 2 diabetes cohort are presented, stratified by quartiles of TyG index. The mean age of the participants was 58.99. Significant differences were observed among the 4 groups in terms of age, race, marital status, BMI, fasting blood glucose, triglycerides, HDL, LDL, and the prevalence of depression.

**Table 1:** Baseline characteristics of participants with type 2 diabetes.

	Total	Q1 (<8.61)	Q2 (8.61–9.03)	Q3 (9.03–9.47)	Q4 (>9.47)	*P* value
Age, yrs	58.99 (0.35)	59.84 (0.60)	60.71 (0.55)	59.28 (0.68)	56.25 (0.64)	<.001
Sex						
Male	1661 (49.90)	426 (50.43)	406 (49.11)	393 (47.61)	436 (52.52)	.540
Female	1564 (50.10)	380 (49.57)	400 (50.89)	414 (52.39)	370 (47.48)	
Race						
Non-Hispanic Black	788 (13.92)	320 (23.91)	187 (13.44)	150 (10.08)	131 (9.29)	<.001
Non-Hispanic White	1182 (63.17)	243 (55.46)	297 (64.79)	337 (67.72)	305 (63.90)	
Mexican American	550 (9.37)	87 (6.20)	119 (7.13)	151 (9.92)	193 (13.83)	
Other Hispanic	366 (5.81)	72 (5.11)	103 (6.68)	94 (5.64)	97 (5.79)	
Other Race	339 (7.73)	84 (9.32)	100 (7.95)	75 (6.64)	80 (7.19)	
Marriage						
Married	2003 (66.20)	477 (63.60)	521 (67.45)	501 (67.93)	504 (65.57)	.021
Never married	293 (8.86)	80 (10.23)	57 (5.16)	67 (8.26)	89 (11.82)	
Divorced	929 (24.94)	249 (26.17)	228 (27.39)	239 (23.80)	213 (22.61)	
BMI, kg/m²						
Normal	453 (12.04)	173 (20.36)	102 (11.33)	108 (10.84)	70 (6.43)	<.001
Overweight	967 (28.26)	242 (31.56)	292 (35.18)	210 (22.47)	223 (24.51)	
Obese	1805 (59.70)	391 (48.08)	412 (53.49)	489 (66.69)	513 (69.06)	
Glucose, mg/dL	146.30 (1.34)	117.26 (1.29)	129.96 (1.54)	140.76 (1.49)	193.99 (3.46)	<.001
TG (mmol/L)	142.66 (2.08)	70.10 (0.93)	111.41 (1.27)	152.51 (1.80)	228.39 (2.99)	<.001
HDL (mmol/L)	49.67 (0.40)	58.24 (1.18)	51.81 (0.64)	47.42 (0.49)	42.14 (0.55)	<.001
LDL (mmol/L)	105.90 (1.01)	95.20 (1.69)	105.18 (1.69)	111.48 (1.93)	110.58 (1.99)	<.001
Smoking						
Never	533 (15.93)	126 (14.17)	129 (15.50)	129 (15.88)	149 (17.99)	.503
Former	1028 (32.79)	250 (31.84)	244 (32.20)	255 (31.93)	279 (35.10)	
Now	1664 (51.28)	430 (53.99)	433 (52.30)	423 (52.19)	378 (46.91)	
Alcohol						
No	1204 (33.91)	295 (32.43)	282 (29.88)	331 (38.57)	296 (34.38)	.058
Moderate	1162 (38.19)	325 (39.47)	299 (41.92)	270 (36.16)	268 (35.50)	
Heavy	368 (12.84)	91 (13.40)	106 (14.20)	84 (11.11)	87 (12.78)	
CHF						
No	3006 (93.60)	737 (93.03)	756 (94.29)	760 (93.64)	753 (93.40)	.869
Yes	219 (6.40)	69 (6.97)	50 (5.71)	47 (6.36)	53 (6.60)	
CHD						
No	2952 (90.38)	740 (93.18)	736 (89.40)	740 (90.18)	736 (89.02)	.133
Yes	273 (9.62)	66 (6.82)	70 (10.60)	67 (9.82)	70 (10.98)	
Depression						
No	2861 (90.23)	749 (94.00)	724 (92.15)	703 (89.60)	685 (85.61)	<.001
Yes	364 (9.77)	57 (6.00)	82 (7.85)	104 (10.40)	121 (14.39)	

Mean (SE) for continuous variables; N(%) for categorical variables; BMI = body mass index, CAD = coronary heart disease, CHF = congestive heart failure, HDL = high-density lipoprotein, LDL = low-density lipoprotein, TG = triglyceride; as demonstrated in Tables 2 and 4.

Furthermore, Table 2 illustrates a higher prevalence of depression in the type 2 diabetes population among obese individuals, females, those who were divorced, nonsmokers, nondrinkers, and those with CHF. Additionally, higher serum triglyceride levels and lower age were also observed to be associated with depression (all *P* < .05).

**Table 2:** Characteristics of participants with type 2 diabetes by depression.

	Total	Without depression	With depression	*P* value
Age, yrs	58.99 (0.35)	59.26 (0.37)	56.48 (0.91)	.005
Sex				
Male	1661 (49.90)	1544 (52.35)	117 (27.25)	<.001
Female	1564 (50.10)	1317 (47.65)	247 (72.75)	
Race				
Non-Hispanic Black	788 (13.92)	702 (13.78)	86 (15.23)	.178
Non-Hispanic White	1182 (63.17)	1054 (63.71)	128 (58.17)	
Mexican American	550 (9.37)	480 (9.21)	70 (10.78)	
Other Hispanic	366 (5.81)	311 (5.48)	55 (8.85)	
Other race	339 (7.73)	314 (7.81)	25 (6.97)	
Marriage				
Married	2003 (66.20)	1838 (68.02)	165 (49.39)	<.001
Never married	293 (8.86)	246 (8.54)	47 (11.89)	
Divorced	929 (24.94)	777 (23.44)	152 (38.72)	
BMI, kg/m²				
Normal	453 (12.04)	416 (12.26)	37 (10.03)	.006
Overweight	967 (28.26)	893 (29.22)	74 (19.36)	
Obese	1805 (59.70)	1552 (58.52)	253 (70.61)	
Glucose, mg/dL	146.30 (1.34)	146.05 (1.42)	148.60 (3.41)	.483
TG (mmol/L)	142.66 (2.08)	140.38 (2.18)	163.69 (4.86)	<.001
HDL (mmol/L)	49.67 (0.40)	49.77 (0.41)	48.72 (0.92)	.273
LDL (mmol/L)	105.90 (1.01)	105.63 (1.08)	108.43 (2.21)	.251
TyG	9.07 (0.02)	9.05 (0.02)	9.25 (0.04)	<.001
Smoking				
Never	533 (15.93)	431 (14.29)	102 (31.06)	<.001
Former	1028 (32.79)	927 (33.07)	101 (30.21)	
Now	1664 (51.28)	1503 (52.64)	161 (38.73)	
Alcohol				
No	1204 (33.91)	1051 (33.26)	153 (39.88)	.002
Moderate	1530 (51.03)	1393 (52.32)	137 (39.04)	
Heavy	491 (15.06)	417 (14.41)	74 (21.08)	
CHF				
No	3006 (93.60)	2680 (93.96)	326 (90.29)	.014
Yes	219 (6.40)	181 (6.04)	38 (9.71)	
CHD				
No	2952 (90.38)	2629 (90.46)	323 (89.72)	.705
Yes	273 (9.62)	232 (9.54)	41 (10.28)	

Mean (SE) for continuous variables; N (%) for categorical variables.

### 3.2. The association between TyG index and depression in individuals with type 2 diabetes

Table 3 presents the results of the multivariable logistic regression analyses. In the fully adjusted model, a significant association was found between the TyG index and the prevalence of depression in the type 2 diabetic population. For every unit increase in the TyG index, the risk of developing depression increased by 54% (OR = 1.54, 95% CI: 1.21–1.95). To further evaluate the robustness of these findings, the TyG index was transformed from a continuous variable to a categorical variable based on quartiles. Compared to the lowest quartile of the TyG index, the second, third, and fourth quartiles were associated with a 28% (OR = 1.28, 95% CI: 0.71–2.32), 73% (OR = 1.73, 95% CI: 1.08–2.76), and 143% (OR = 2.43, 95% CI: 1.52–3.89) increased risk of developing depression, respectively. These findings provide additional evidence of the association between the TyG index and the prevalence of depression in individuals with type 2 diabetes.

**Table 3:** Association between TyG index and depression in population with type 2 diabetes.

	Model I		Model II		Model III	
	95% CI	*P* value	95% CI	*P* value	95% CI	*P* value
TyG	1.60 (1.29, 1.99)	<.001	1.64 (1.30, 2.07)	<.001	1.54 (1.21, 1.95)	<.001
TyG index quartiles						
Q1	Ref		Ref		Ref	
Q2	1.34 (0.77, 2.32)	.303	1.36 (0.76, 2.43)	.297	1.28 (0.71, 2.32)	.414
Q3	1.82 (1.15, 2.88)	.011	1.86 (1.17, 2.96)	.010	1.73 (1.08, 2.76)	.023
Q4	2.64 (1.69, 4.12)	<.001	2.77 (1.74, 4.42)	<.001	2.43 (1.52, 3.89)	<.001
*P* for trend	<.001		<.001		<.001	

Model I: no adjusted.

Model II: adjust for age, sex, marriage, and race.

Model III: adjusted for age, sex, marriage, race, body mass index (BMI), smoking, alcohol, congestive heart failure (CHF), and coronary heart disease (CAD).

### 3.3. Dose–response relationship

**Figure 2.**
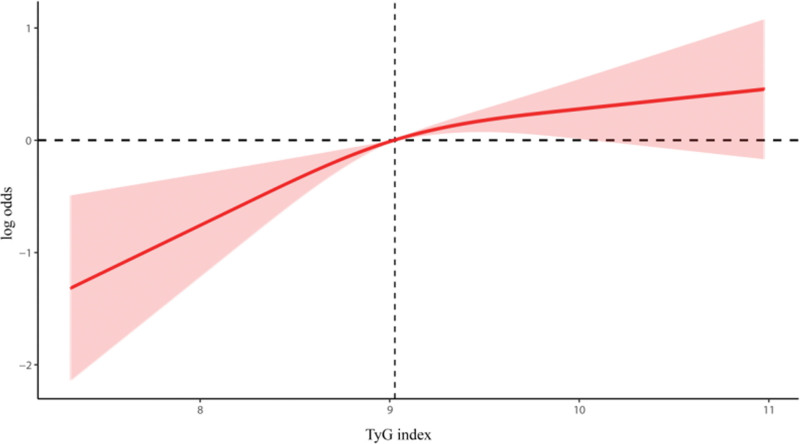
Dose–response relationship of TyG index and depression in patients with type 2 diabetes. TyG = triglyceride-glucose.

In order to explore the potentially nonlinear relationship between the TyG index and the incidence of depression in the type 2 diabetic population, RCS were employed. This enabled us to depict the dose–response association between these 2 factors. Figure 2 shows the results of this analyses, with a linear rather than a nonlinear relationship between the TyG index and the incidence of depression in the type 2 diabetes population (*P* Overall < .001; *P* Nonlinear = .120).

### 3.4. Subgroup analyses

In the final phase of this study, subgroup analyses were performed to explore the relationship between the TyG index and depression in individuals with type 2 diabetes mellitus with different characteristics. The potential moderating effects of age, gender, marital status, BMI, smoking, alcohol consumption, CHF, and CAD on this relationship were examined using interaction effects (Table 4). The results revealed that the association between the TyG index and depression remained robust across different age groups, genders, BMI, smoking habits, alcohol consumption patterns, and the presence of CHF or CAD. Notably, in individuals with normal weight, there was a significant association between a higher TyG index and a reduced risk of depression, although the interaction effect with BMI was not statistically significant.

**Table 4:** Subgroup analyses of TyG index and depression in patients with type 2 diabetes.

Subgroup	Q1	Q2	Q3	Q4	*P* for trend	*P* for interactions
Age, yrs						
≥60	Ref	1.40 (0.65,3.02)	1.43 (0.79,2.56)	3.26 (1.82,5.85)	<.001	.127
<60	Ref	1.14 (0.52,2.51)	1.89 (0.96,3.74)	1.88 (1.02,3.47)	.009	
Sex						
Male	Ref	2.43 (1.05,5.62)	1.85 (0.77,4.47)	4.54 (2.15,9.62)	<.001	.229
Female	Ref	0.99 (0.48,2.03)	1.65 (0.90,3.01)	1.91 (1.02,3.57)	.012	
BMI, kg/m²						
Normal	Ref	1.92 (0.57,6.44)	0.58 (0.14,2.38)	0.55 (0.12,2.39)	.374	.090
Over weight	Ref	1.91 (0.51,7.11)	2.67 (0.80,8.88)	3.20 (1.01,10.17)	.028	
Obese	Ref	1.05 (0.55,1.99)	1.83 (1.00,3.38)	2.65 (1.40,5.02)	<.001	
Smoking						
Never	Ref	1.17 (0.56,2.46)	1.86 (0.93,3.75)	2.44 (1.21,4.92)	.003	.091
Former	Ref	2.36 (0.90,6.23)	3.20 (1.48,6.91)	6.09 (2.99,12.40)	<.001	
Now	Ref	0.76 (0.31,1.88)	0.88 (0.35,2.21)	1.05 (0.46,2.39)	.760	
Alcohol						
No	Ref	1.33 (0.51,3.47)	2.46 (0.94,6.41)	3.00 (1.21,7.44)	.004	.818
Moderate	Ref	1.17 (0.52,2.60)	1.26 (0.68,2.32)	2.16 (1.11,4.20)	.017	
Heavy	Ref	1.57 (0.46,5.34)	1.71 (0.57,5.14)	2.58 (1.07,6.24)	.031	
CHF						
No	Ref	1.17 (0.61,2.21)	1.65 (1.01,2.68)	2.31 (1.40,3.81)	<.001	.624
Yes	Ref	3.53 (0.69,18.05)	3.06 (0.59,15.79)	7.30 (1.57,34.03)	.009	
CHD						
No	Ref	1.35 (0.71,2.56)	1.73 (1.05,2.86)	2.30 (1.38,3.84)	<.001	.376
Yes	Ref	0.89 (0.16,5.03)	2.00 (0.48,8.22)	5.49 (1.39,21.74)	.007	

All variables were adjusted in the analyses except for the stratified own variable; *P* for interactions were calculated using the likelihood ratio test.

## 4. Discussion

This study utilized a cross-sectional design and included a nationally representative sample of 3225 individuals with type 2 diabetes mellitus. The findings of the study provide compelling evidence of a significant independent positive association between the TyG index and depression. This research is particularly noteworthy as it is the first to establish a link between the TyG index and depression specifically in the type 2 diabetic population. Additionally, the study described a dose–response relationship between the TyG index and depression among individuals with type 2 diabetes, further supporting the robustness of the association. Furthermore, the study findings showed consistent associations between the TyG index and depression across various subgroups stratified by age, gender, smoking, alcohol consumption, CHF, and CAD. This suggests that the association between the TyG index and depression holds true regardless of these demographic and clinical factors.

Previous studies have provided evidence that individuals with comorbid type 2 diabetes and depression have distinct characteristics compared to those with depression alone. The presence of type 2 diabetes nearly doubles the risk of developing depression.^[23]^ Furthermore, comorbid depression in individuals with type 2 diabetes can contribute to a range of complications associated with both conditions. In a 5-year follow-up cohort study, researchers found that severe depression in individuals with type 2 diabetes was associated with a higher risk of clinically significant microvascular and macrovascular complications.^[24]^ Moreover, studies have also examined the relationship between comorbid depression and dementia in individuals with type 2 diabetes.^[23]^ The results indicate that depression significantly increases the risk of developing dementia among individuals with type 2 diabetes.^[25]^ Therefore, depression in individuals with type 2 diabetes requires particular attention. Currently, there is limited research on the risk of depression in people with type 2 diabetes. However, there are some noteworthy studies that shed light on the subject. One study conducted among U.S. adults investigated the association between the neutrophil-to-lymphocyte ratio and the incidence of depression in patients with type 2 diabetes. In a national survey of type 2 diabetics, the study found that the higher the neutrophil-to-lymphocyte ratio, the greater the risk of depression.^[26]^ Similarly, a Chinese study examined the relationship between serum high-sensitivity C-reactive protein (hs-CRP) levels and the risk of depression in women with type 2 diabetes. The results showed that elevated hs-CRP levels were associated with an increased risk of depression in women.^[27]^ However, no significant association was observed in men. In addition, a study conducted in a Japanese community showed a higher prevalence of depression in community-based type 2 diabetes patients who were moderate to heavy smokers.^[28]^ Consistent with previous findings, our study found that the higher the TyG index, the higher the risk of depression in the type 2 diabetic patients, even after accounting for various confounding factors. These findings will undoubtedly help to fill the gap in the literature regarding the risk of depression in this particular population.

The exact mechanism underlying the relationship between the TyG index and depression in individuals with type 2 diabetes is not yet fully understood. However, there are some potential mechanisms that have been proposed based on previous research. The TyG index is considered as an indicator of insulin resistance and has been associated with various metabolic and physiological alterations.^[29–31]^ These alterations include inflammation, hormonal disruptions, and lipid metabolic disturbances, which have all been found to be involved in the development and manifestation of depressive symptoms in individuals with type 2 diabetes.^[32–34]^

Despite being a large cross-sectional study that considers potential confounding factors, it is important to acknowledge the limitations of this research. Firstly, the cross-sectional design of this study limits our ability to establish a causal relationship between the TyG index and the incidence of depression in a type 2 diabetes population. While we have observed an association, further longitudinal or interventional studies are needed to determine the directionality and causal nature of this relationship. Secondly, although efforts were made to control for confounding factors, there may be other unmeasured confounders that were not included in this study. These unmeasured variables could potentially affect the observed association between the TyG index and depression and may influence the generalizability of our conclusions. Lastly, it is important to note that this study focused solely on the population in the United States. Therefore, caution should be exercised when attempting to generalize these findings to other populations or countries. Further large-scale studies conducted in diverse populations are needed to obtain more universally applicable conclusions.

## 5. Conclusion

In conclusion, the results of this study suggest a significant independent association between higher TyG index and increased prevalence of depression in patients with type 2 diabetes.

## Acknowledgments

We much appreciate School of management of Beijing University of Chinese Medicine, School of Traditional Chinese Medicine of Beijing University of Chinese Medicine, Dongzhimen Hospital Beijing University of Chinese Medicine and General Medicine Department of Emergency General Hospital for providing support on the study.

## Author contributions

**Conceptualization:** Jiaju Ren.

**Data curation:** Jiaju Ren, Cheng Lv, Jia Wang.

**Formal analysis:** Jiaju Ren.

**Methodology:** Jiaju Ren.

**Supervision:** Jiaju Ren, Jia Wang.

**Validation:** Jiaju Ren.

**Visualization:** Jiaju Ren.

**Writing – original draft:** Jiaju Ren, Cheng Lv, Jia Wang.

**Writing – review & editing:** Jiaju Ren.
